# Case of Death after Parturition

**Published:** 1844-11-30

**Authors:** 


### RECORD OF MEDICAL SCIENCE.

### CASE OF DEATH AFTER PARTURITION.

BY DR. BOND.

*April 8th*, 1844. At 10 P. M., he was requested to visit Mrs. R——, and was informed by the messenger that she was affected with cholic. Mrs. R. had a good form and constitution, and enjoyed good health until this attack. She is twenty-eight years of age; was married on the 4th of last October; had never menstruated after marriage; supposed that she became pregnant within one week after that event, and expected to be confined in the beginning of the second week in July. About noon to-day she was reaching over a flour barrel that stood end-wise, to pick up something behind it, in doing which she felt something give way, and was attacked with pain in that part of the abdomen where she came in contact with the sharp edge of the barrel. The pain continued all the afternoon and evening, and was supposed by her female attendants to be colic, and, in consequence, ginger tea and other domestic remedies had been administered. But the pain became worse in the evening, and when the doctor visited her, she evidently had labor pains, but no stools or discharge; he directed her an opiate, rest, and a cool regimen, with orders to send for him if she was not shortly relieved. At 11½ o'clock, P. M. he was sent for again. The pains had increased, and the os uteri had become relaxed, and more than half dilated. The pains were more distressing than usual in such cases, and she was inclined to keep her hand applied to that part of the abdomen which had come in contact with the barrel. The membranes were very strong, and continued unbroken, until a loose sac of water presented externally, and when broken, there was a gush of a large quantity of liquor amnii. The delivery of the child followed almost immediately afterwards—April 9th at 2¼ A. M. Soon after this, he began to use the ordinary means to promote the delivery of the placenta, which he continued to do for three quarters of an hour without any effect. There was no sign of its coming; there was neither any expulsive pain, nor the least appearance of any coloured discharge. He then gave twenty grains of ergot, and after a quarter of an hour, twenty grains more. At the end of ten or fifteen minutes more, no uterine contraction had occurred,—at least he discovered none, either by questioning the patient or by watching her motions. It is proper to observe, that the ergot, although procured at the time from one of the best shops, was almost entirely destitute of the peculiar odour which he had generally observed to belong to that which has been most effective.

The uterus was so low that he could easily pass his finger within its neck which was soft and relaxed; but above that, the uterus seemed to have assumed something like an hour-glass contraction, so low down towards the neck, as to embrace in the upper chamber nearly or quite all of the placenta. He then prepared to introduce his hand, without waiting longer for the ergot to act. The cord was ten inches long, very large, and firm for that period of gestation, except for a short distance near its insertion into the placenta, where it was very slender. In his attempts to examine the condition of the uterus and to deliver the placenta, the cord was torn from its attachment. This occurred only a short time before the introduction of the hand. The os externum and the os cervix uteri were so relaxed, that the hand passed with ease until it reached the above mentioned contraction. This was so rigid as to occasion some delay. During the process of introducing the hand, pretty strong uterine contraction came on, which increased the delay. Whether this was the effect of the ergot, or of irritation of the hand, is doubtful;—probably it was partly owing to each. Upon passing the hand through the stricture, into the chamber containing the placenta, it was found that this was detached from the uterus in no part, so far as could be perceived. Up to this period there was no sanguineous discharge, except a very little, which was supposed to proceed from the separation of the cord. The doctor proceeded slowly and carefully to detach the placenta, which adhered so firmly as to make the operation tedious. After the whole of the placenta was separated, he found a strong membranous attachment to one part of the fundus, which he could not overcome. It was of limited extent, being less than an inch. He used as strong traction as he thought could be done without endangering a laceration of the uterus. When pulling it, it seemed to yield a little, but upon examination, there was no sign of its being detached. It occurred to him that, possibly, there might be an hour-glass contraction, embracing a small part of the placenta and membranes, although he could perceive no rounded edge and depression around the adhesion such as he should expect in that case; but to determine this, he applied the other hand to the abdomen, and this examination entirely satisfied him that such could not be the case. He then separated the placenta from this adhering membrane, and withdrew it. The membrane, which felt like a ligament, had such remarkable strength, that the separation was not easily accomplished. The operation occupied three quarters of an hour, and was attended with very little hæmorrhage.

The child lived twelve hours. The fœtal end of the cord, as before observed, was very thick, and he tied it, as he thought, very carefully: but in a little while he discovered that, in proportion to the size of the child, there was considerable hæmorrhage, such as might have destroyed it, had it not been discovered and arrested. It had not, however, produced, any signs of anæmia.

April 10. Last evening the patient began to be uneasy, and feverish—a cathartic of senna, manna, and sulph. magnesiæ, in divided doses was directed. It operated sufficiently during the night, but the febrile symptoms and uneasiness were not mitigated. Tongue a little coated; pulse 110 to 120; some tenderness in the left iliac region; the uneasiness in the head increased, In the afternoon the symptoms all aggravated; no lochial discharge. Directed the neutral mixture; v. s. oz. xxiv; fomentations. The blood drawn exhibited a thick buffy coat, and small cupped coagulum.

April 11. Head much relieved since the v. s. but the patient had a very uncomfortable night; tongue has a yellowish white coat, thickest in the middle; had a severe chill this morning at 7 o'clock; pulse 120 to 130. After the applications of the fomentation, a copious sanguinous discharge from the vagina came on, which it was thought desirable to promote, therefore the v. s. was not repeated nor leeches resorted to this morning as would otherwise have been done. Directed cal. ipecac and opium. At 5 P. M. pulse 102, at 11 P. M. 82; all the symptoms mitigated. The pain in the abdomen, last night and today, which was almost entirely confined to the iliac region, was intermittent like after pains.

April 12. In the morning found her more comfortable in every respect; pulse 80 to 90. No tympanitis or swelling of the abdomen, and the tenderness is chiefly confined to the part before mentioned; sanguineous discharge has ceased, and the vaginal discharge has become offensive; some confusion of the head in the night and this morning. When visited at 5 P. M. the patient was found to have become worse soon after the morning visit; great increase of febrile excitement and of tenderness of the abdomen; thirst great; began to be tympanitic. Upon examination found some shreds of membrane in the neck of the uterus, which were removed; one part, however, extended into the uterus, which could easily be seized hold of, but adhered too firmly to be extracted without applying more force than could be done with safety. Sometime afterward it came away and the offensive discharge ceased.

April 13. She began in the night to belch up whatever she drank; pulse very variable; tympanitis increasing, as well as the distress and tenderness in the abdomen. Dr. Meigs saw the patient twice to-day, but it is unnecessary to detail the treatment which had no influence in controlling the disease. The discharges from the stomach became more and more dark,until they closely resembled coffee grounds. The patient grew rapidly worse, and died next morning, April 14th, at 4 A. M., about 98 hours after delivery.

Autopsy, 29 hours after death. The operation was performed by Dr. Goddard, in the presence of Drs. Huston and Bond; and this account of it is chiefly taken from Dr. Goddard's notes. Abdomen alone examined. On opening the peritoneal cavity, a quantity of fœtid gas escaped, which occurrence Dr. G. observed he had never met with, except in cases of perforation of the intestines, or as a result of putrefactive fermentation. That part of the peritoneum, where this gas was contained, was dry and glossy, as if it had been hung up in the open air; colon much distended and very low in the abdomen. Upon carefully raising the omentum majus, the whole of which was gangrenous and fœtid, a black mass, about two inches long and three quarters of an inch wide, was brought into view, one end of which passed into an opening in the fundus of the uterus. At first view this looked like a coagulum of blood, which in some parts had began to be organized, but which, being drawn out and washed, proved to be a portion of the fœtal membranes, with some remains of placental fibres, and the debris of a coagulum of blood—the whole being in an advanced state of putrefaction. The uterus was next examined, and found to be lacerated at the left extremity of the fundus, above and very near the junction of the Fallopian tube. The rupture in the peritoneum was small—about three-fourths of an inch in length, and entirely filled up by the membrane before alluded to. The laceration of the proper substance of the uterus was greater, and its edges were covered with a coagulum partially organized. The lining of the uterine cavity elsewhere looked well. The peritoneum showed traces of high inflammation in several parts—about the stomach, among the small intestines and especially at the pelvic region, but appearing comparatively healthy at the anterior portions of the abdomen. There was very little effusion into the peritoneal cavity—there was a small quantity of blackish fluid, with flakes of pus floating in it, in the most dependant portions of the abdominal cavity.

There is, therefore, reason to believe that, when the laceration took place, it was chiefly in the uterine substance, the peritoneum resisting; that the unbroken membranes protruded, like a hernia, through the aperture into the abdominal cavity; that the contraction of the uterus, after the delivery of the child, closed so firmly upon these as to strangulate them, and to resist the efforts to withdraw them, during the operation for extracting the placenta. This examination explains the difficulty attendant upon the delivery of the secundines; for although the placenta was in no part detached from the uterus when the hand was first introduced, still there did not appear to be any preternatural adhesions or growing of the placenta to the uterus; but the chief difficulty arose from the extraordinary strength of the fœtal membranes, and their inextricable confinement. The revelations of this examination, were especially gratifying to the doctor, as he had apprehensions before this, that although the labor was brought on by an accident, the febrile and inflammatory symptoms and the fatal termination, might have been the consequence of the operation of extraction; although he had proceeded with such caution, that he was satisfied it could have resulted only from the irritation of the protracted operation, and not from any laceration, or other violence done to the part.

Dr. Bell remarked, that the case just detailed was certainly a highly interesting one. From the known experience and caution of the gentleman by whom they were performed, there could be no doubt but that the measures pursued, with the view of detaching and bringing away the placenta, were conducted with due care; there was, however, he was convinced, far too much anxiety, in general, entertained in regard to tbe retention of the placenta, or any portion of it over a certain period, within the cavity of the uterus, and not unfrequently, he apprehended, injury has been inflicted upon that organ by the efforts made to bring away the placenta, especially by the introduction of the hand; and when a preternatural adhesion of the placenta to the surface of the uterus exists, it is to be feared that its forcible separation may be the cause of some serious lesion, even laceration or rupture of the walls of the uterus. If no immediate ill consequences result from the retention of the placenta, would it not be better in every case to wait, until it is thrown off by the contraction of the uterus, which may be accelerated perhaps by friction, slight traction of the cord and similar means. Even when the retention results from preternatural adhesion of some portion of the placenta, if it cannot be entirely detached without the exertion of more than the slightest degree of force, it will certainly be separated after a time, and discharged with the lochia. On the experienced and careful obstetrician the importance of caution in regard to this subject need not be enforced, but the young, and inexperienced practitioner should be as much as possible dissuaded from every species of interference, in any case of labor, in which it can, with safety to the patient, be possibly avoided. We are convinced, that in the practice of midwifery much less danger is liable to result from patiently waiting for the operations of nature, which, though often tardy, are in the end seldom inefficient, than from too rash and early a resort to instrumental assistance.

Dr. Moehring would inquire, whether any of the Fellows present had ever known injury to result from waiting until the placenta was expelled by the action of the uterus—the usual means being in the meantime resorted to to induce its complete contraction. Several of the continental writers on obstetrics forbid all interference, excepting in cases where profuse hæmorrhage or other alarming symptoms occur—he had himself allowed the placenta to remain for a day or even longer, when it came away without any injury or suffering to the female resulting. He believed that the danger from retention of the placenta for many hours after the birth of the child was overrated by many practitioners, and that this often caused a too early and improper interference in order to effect its removal.

Dr. Condie remarked, that he had supposed the rules in regard to the management of the placenta to be well settled; he was not aware that any important difference of opinion existed on this subject among the practitioners of midwifery in this country. The direction given by the late Professor James was, excepting in cases in which the occurrence of hæmorrhage or other alarming symptoms demanded its earlier removal, to wait for at least one hour after the delivery of the child for the expulsion of the secundines, when, if this did not occur, to introduce the hand and remove them—but in no case to leave the patient until the placenta was brought away, and the uterus well contracted. This direction, he is convinced, is a very judicious one. A very common cause of retained placenta, being an hour glass contraction of the uterus, some have even advised that in all cases, when, after the delivery of the child, the placenta is found not to come away, upon gentle traction of the cord and brisk friction over the region of the uterus being resorted to, that its delivery should be effected by the hand, inasmuch as the partial contraction is then much more easily overcome than it will be at a later period. The rule being understood, that whenever the placenta is not expelled by the action of the uterus within a short period after the birth of the child, manual interference is required, the precise time it is proper to wait in each case must be left to the judgment of the practitioner. In some, it may be necessary to bring away the placenta immediately, while in others, an hour or two may, with great propriety, be allowed to elapse before this is done. In general, however, he believed that the sooner the placenta is delivered the better. The practice of leaving a female with a retained placenta, and waiting many hours or even days for its discharge, cannot be too strongly reprobated—it being attended always with the utmost risk to the patient. The doctor stated that he had known, when this practice had been pursued, the patient to become exhausted by an almost continued hæmorrhage, which ceased almost immediately after the placenta was removed. In other instances, when the entire placenta, or a considerable portion of it has been allowed to remain and become putrid—all the symptoms of a low typhoid fever ensued, under which the patients sunk. By more than one of the recent writers on obstetrics, the danger of allowing any portion of the secundines, or coagula of blood to become putrid in the cavity of the uterus, is very strongly insisted upon, and it is even recommended that injectious of some tepid fluid should be resorted to, in order to wash out the uterus and vagina. The origin of one of the most unmanageable forms of puerperal metritis, has been ascribed to the absorption, by the open mouths of the uterine veins, of putrid matter, resulting from the retention and decomposition within the uterus, of portions of the after birth, or coagula.

Dr. Bell did not doubt that cases might occur in which the prompt removal of the placenta would be called for—but he was persuaded, that in the generality of cases, less danger will be liable to result from waiting until it is thrown off by the action of the ut rus, than from the injury which must, to a greater or less extent, be inflicted upon this organ, already predisposed to disease, by the introduction into its cavity of the hand and the manipulations necessary to separate, grasp, and bring away the placenta. In some cases, the connection of the latter with the uterus is so intimate, that, we are told, much time and some degree of force are required completely to separate it. Is it possible to suppose that the delicate texture of the uterus can escape in such cases without injury? It is said that there is danger of hæmorrhage so long as the placenta remains undelivered. This may be true in certain cases, but does it not depend upon the very same circumstance which causes the after birth to be retained; namely, the imperfect contracting of the uterus; and will not a resort to those means which tend to excite contraction of the uterns, enables us to control the hæmorrhage, and at the same time cause the expulsion of the placenta? The supposition that puerperal or any other malignant form of fever will be produced by the retention and putrefaction of the placenta is extremely problematical. It is not uncommon for a portion of membrane or coagula of blood, subsequent to parturition, to become putrid within the uterus, and be discharged in this state with the lochia—and yet we know, that puerperal metritis or typhus fever is of comparatively rare occurrence. The doctor remarked, that it was not his desire to advocate a universal rule of non interference in cases of retained placenta, but to urge the necessity of trusting in these, as in all the ordinary cases, and throughout every stage of labour, much to the operation of the natural powers, and of avoiding all unnecessary and too early interference.

Dr. Warrington had always acted upon the rule of not leaving his patient until the placenta was expelled, and provided this does not take place spontaneously, within a reasonable period after the delivery of the child, to introduce the hand and extract it: it had nevertheless fallen to his lot to observe a few cases, in which the placenta was allowed to remain for many hours—in one for twelve hours—without the occurrence of any injury or suffering on the part of the patient. He believes the safest rule to be, however, to wait for an hour, and if the placenta is not then expelled—frictions over the hypogastrium, gentle traction of the cord, and perhaps the administration of a dose of ergot, having in the meantime been resorted to---to deliver by the hand.---*Trans. College of Physicians.*

